# Retrospective Epidemiological Study of Burn Injuries in 1717 Pediatric Patients: 10 Years Analysis of Hospital Data in Iran

**Published:** 2018-04

**Authors:** Jafar KAZEMZADEH, Reza VAGHARDOOST, Mostafa DAHMARDEHEI, Soheila RABIEPOOR, Ramyar FARZAN, Ali ASGHAR KHEIRI, Rahman KHOSRAVY

**Affiliations:** 1. Dept. of Reconstructive and Burn Surgery, Faculty of Medicine, Urmia University of Medical Sciences, Urmia, Iran; 2. Burn Research Center & Dept. of Plastic and Reconstructive Surgery, Faculty of Medicine, Iran University of Medical Sciences, Tehran, Iran; 3. Reproductive Health Research Center & Midwifery Dept., Faculty of Nursing & Midwifery, Urmia University of Medical Sciences, Urmia, Iran; 4. Dept. of Reconstructive and Burn Surgery, Faculty of Medicine, Guilan University of Medical Sciences, Rasht, Iran; 5. Dept. of Reconstructive and Burn Surgery, Faculty of Medicine, Tabriz University of Medical Science, Tabriz, Iran; 6. Dept. of Pediatric Surgery, Faculty of Medicine, Urmia University of Medical Sciences, Urmia, Iran

**Keywords:** Burn, Injury, Epidemiology, Pediatric

## Abstract

**Background:**

Burn injuries are considered an important preventable cause of injuries in children, and it still produces significant death in Iran. This study investigated the causes and severity of burns in patients.

**Methods:**

This study was retrospective descriptive study of children-burn injury in a referral Burn Care Center in Tehran, Iran during a ten-year period since 2005 to 2014. Data collection have been facilitated by using a specially designed checklist. The subjects included 1717 consecutive patients with various causes of burn injury. Data were analyzed applying descriptive statistics, one-way ANOVA, Chi-square. *P*-values less than 0.05 were considered significant.

**Results:**

The patients’ mean age was 4.11 ± 3.42 yr. The mean hospitalization period was 11.15 ± 8.37 d. The grade of burn was 2 in 1292 (75.2%) patients. Among the children-burn patients, 59.9% suffered from <20% of total body surface area burn. Most affected part of the body was trunk 762 (44.4%). Overall, 1256 patients (73.2%) suffered from hot liquid burns. Burns mortality rate for this study was identified 8.1% (N=3).

**Conclusion:**

The majority of the patients were male with a male to female ratio of 1.7:1. Most patients were in the 2–4 yr age group, with most of the injuries occurring in boys under the age of 5 yr old. It is the child’s natural curiosity and inability to understand that special things are dangerous to them, which leads to burning injury. Most affected part of the body was trunk and 1256 patients (73.2%) suffered from hot liquid burns.

## Introduction

Burn injuries represent a significant public health problem across the world (1). About 2.5 million people are burned annually in the United States (2), also in Iran about 725 thousand burns occur every year (3). Burn injuries in the world after the accident falls and violence is the fourth most common type of trauma (4). Children in third world countries due to poverty and disease are at greater risk for morbidity and mortality (5). Moreover, they are at a high risk of burn injuries because of too many reasons, such their natural curiosity, impulsiveness, less rapid perception of dangerous situations and a limited ability to react promptly and properly in dangerous situations (6).

Burn is the third most common cause of mortality in children and teens (7). Mortality from pediatric burns is majority pronounced in low- and middle-income countries (LMICs), otherwise 3.4 burn-related mortality per 100000 children in LMICs compared to 0.5 mortality per 100000 in high-income countries (6, 8).

Burn injury is a major health concern in the pediatric age group in Iran and also after traffic accident, it is the second cause of mortality in this group (9, 10); however, burns can be easily prevented in children (11).

The epidemiology of burn injury is diverse across the world, also within a country due to differences in the cultural and socioeconomic factors, so we identified the socio-demographic characteristics of children-burn patients, to investigate the causes and severity of burns and to describe the associations of the problem in children-burn patients admitted to the Motahari Hospital over a period of 10 yr.

## Materials and Methods

### Setting

This retrospective descriptive study was conducted in Motahari Referral Hospital, Tehran, Iran during a ten-year period since 2005 to 2014. This hospital represents a reference center across the region and this burns unit is the only regional referral center for all burn injuries in the city. Motahari Burn and Reconstruction Center are one of the few highly equipped tertiary burn centers in Iran, providing care to burned patients from the province of Tehran and too complicated cases referred from other centers across Iran. Ethics Committee of the hospital approved the study.

### Design

We conducted a retrospective hospital-based analytical study in Motahari Hospital in Tehran, Iran. This study evaluated 1717 document for children with different burn injuries, to identify the epidemiology of burns in this hospital. Children were divided into four groups based on their ages: ≤1 year, 2–4 yr, and 5–9 yr and 10–15 yr. Moreover, the cause of burns was divided into three groups based on burn cause: hot liquids (water, milk, etc.) and dry heat (electricity and flame explosion, etc.).

### Selection of study participants

All children with a burn-related injury were included in this study. There were 1717 patients, ranging from infancy to fifteen years, who consented, assented and met the criteria for burns admission in the hospital, were included. No patient was excluded from the study. All available cases were retrieved without any exclusion.

### Data collection

Overall, 1717 patients in a period of 10 yr were subjected to a checklist to obtain the following data: age, sex, brief description of the event, site affected the Total Burned Surface Area (TBSA) incurred, dates of admission and discharge, Outcome was recorded as patient survival or death. Other variables were specifically on what circumstance, how long the hospital stays.

One trained research assistant, with the authors supervising, recorded data by use of a well-designed checklist of the burns patients. The trained research assistant was nurse, working in burn and the surgical ward with experience in burns management.

The checklist was collected by reviewing medical records at the burn unit of the hospital. All cases were selected for study but documents were excluded, if more than 20% of the required data were incomplete.

### Data analysis

SPSS (Chicago, IL, USA) release ver. 22.0, was used for data analysis. Descriptive statistics (means with standard deviations or frequency distribution) of sociodemographic variables were computed.

## Results

Generally, 1717 burn-children were evaluated. The majority of the patients (n=1081, 63%) were male as compared to female (n=636, 37%) with a male to female ratio of 1.7:1. The mean age was 4.11 ± 3.42 yr (range, less than 1 to 15 yr old). Most patients (45.7%) were in the 2–4 yr age group. The mean hospitalization period was 11.15 ± 8.37 (1–63) d. The majority of the children-burn patients spent 2 wk in the hospital 602 (35.1%). The most grade of burn was II 1292 (75.2%). Among the children-burn patients, 1028 (59.9%) suffered from < 20% of total body surface area burn, and the rest 689 (40.1%) > 20% of total body surface area burn. The body surface burn percentage mean was 21.23 ± 11.75. Most affected part of the body was trunk 762 (44.4%), next was feet 639 (37%). As most parts of children-burn patient’s body affected by burn-injury, so the frequency was more than 1717 patients. Based on the classification of cause of burn in our patients, 1256 patients (73.2%) suffered from hot liquid burns. Main causes of hot liquid burn and steams and those due to dry heat and fire in children were respectively: boiling water in 947 (55.15) cases and flame in 89 (5.2%) patients. Burns with non-flammable materials such as petroleum 114 (6.6%) cases and acid 91 (5.3%) cases, hot food burns in 216 (12.6%) cases and other hot bodies in 2 (0.1%) patients. In 4 (0/3%) cases were self-immolation, 3 (0/2%) was other-immolation. Burns mortality rate for our study was identified 4.5% (78). 1464 subjects (85.3%) were discharged by physicians, 169 (9.8%) were discharged by self, and 6 subjects (0.3%) were referred to other health-care centers (Table 1). The duration of hospital stay and death was high in 10–15 yr age group, and 5–9 yr age group, respectively (Table 2).

**Table 1:** Distribution of the children-burn patients (n=1717) by socio-demographic characteristics, hospital stay, grade of burn, total body surface area (TBSA) burn, and parts of the body

***Variables***	***Frequency***	***Percentage***
Age(yr)		
≤1	375	21.8
2–4	785	45.7
5–9	377	22.0
10–15	180	10.5
Sex		
Male	1081	63
Female	636	37
The duration of the hospital stay		
1 day	106	6.2
2 – 3 d	145	8.4
3 – 5 d	196	11.4
1 week	211	12.3
2 wk	602	35.1
3 wk	281	16.4
> 3 wk	176	10.3
Grade		
I	20	1.2
II	1292	75.2
III	405	23.6
% of TBSA burn		
1–10	336	19.6
11–20	692	40.3
21–30	335	19.5
31–40	174	10.1
41–50	99	5.8
51–60	27	1.6
61–70	27	1.6
71–80	12	0.7
81–90	8	0.5
91–100	7	0.4
Area		
Head/face/neck	389	22.9
Trunk	762	44.4
Hands	577	32.9
Feet	639	37.0
Wrist and Ankle	473	27.9
Multiple organs	535	31.2
Inhalation	38	2.4
Cause of burn		
Hot liquids	1256	73.2
Dry heat	461	26.8

**Table 2:** Distribution of the children-burn patients by age groups, hospital stay, degree of burn, sex, and outcome

***Age groups (yr)***	***Hospital stay Mean ± SD***	***Sex N (%)***		***Outcome N (%)***	
		**male**	**female**	**survival**	**Death**
≤1	9.15 ± 7.07	240(64)	135(36)	360(96)	15(4.0)
2–4	11.02 ± 7.70	486(61.9)	299(38.1)	760(96.8)	25(3.2)
5–9	12.43 ± 9.12	233(61.8)	144(38.2)	351(93.1)	26(6.9)
10–15	13.18 ± 10.84	122(67.8)	58(32.2)	168(93.3)	12(6.7)

There were 1028 (59.9%) patients with TBSA less than 20%. Moreover, about 10.9% of deaths occurred in patients with TBSA over 20%. Mean of age and hospital stay were high in children-burn patients with TBSA more than 20% (Table 3).

**Table 3:** Distribution of pediatric burn by TBSA%, frequency, age and hospital stay

***TBSA%***	***Frequency (%)***	***Age Mean ± SD***	***Hospital stay Mean ± SD***	***Outcome (%)***	
				**Survival**	**Death**
≤ 20	1028 (59.9)	3.88 ± 3.30	8.41 ± 6.01	1025(99.7)	3(0.3)
More than 20	689 (40.1)	4.46 ± 3.56	15.24 ± 9.64	614(89.1)	75(10.9)

The majority frequency was related to the burns caused by boiling water (54.7%). In addition, 24.2% of the burns were due to other causes, such petroleum, self-immolation, other-immolation, and domestic gas. Most mortality rates was related to other cause of burns (Table 4).

**Table 4:** Distribution of pediatric burn by cause of burn, age and hospital stay and outcome

***Cause of burn***	***Frequency (%)***	***Age Mean ± SD***	***Hospital stay Mean ± SD***	***Outcome (%)***	
				**Survival**	**Death**
Scald	939(54.7)	3.13 ± 2.64	10.33 ± 7.12	919(97.9)	20(2.1)
Hot liquid	307(17.9)	3.97 ± 3.17	10.57 ± 7.51	298(97.1)	9(2.9)
Electricity	55(3.2)	6.78 ± 7.19	13.45 ± 13.68	54(98.2)	1(1.8)
Other	416(24.2)	6.08 ± 3.98	13.11 ± 10.16	368(88.5)	48(11.5)

## Discussion

In the Western countries, due to safety measures and preventive programs, the incidence of children-burn injuries is decreasing; but in developing countries, children burns injuries continue to be an important cause of morbidity and mortality (12). Therefore, we evaluated 1717 children burn injuries to found the epidemiologic variables in this population.

Majority of the pediatrics in the present study were male. Patterns of fatal scald burns were reported more in male (66.7%) (13). Overall, 816 cases of burns were found in 57.5% male and 43.5% female (14). The gender ratio was approximated 3: 2 (Male: Female) (15). Overall, 610 male and 404 female children under the age of 15 yr, were burned (10). A predominance of burns was showed among boys compared to girls (16). The personal and behavioral characteristics feature of male child susceptible them for more burn-injuries. The fact that boys have a tendency towards being more vivacious, active and curious by nature may cause these results. Due to the more incidences of burns in boys, more attention is required in this sex group.

In the current study, mean age was 4.11 ± 3.42 yr. Mean age of the 1215 cases, was 3.74 ± 4.3 yr (15). Besides, the mean age of children-burn patients was 4 ± 3/7 yr (17). Another hand, in Cairo University Hospital-Egypt, the average age of the children-burn patients, was 4 yr old (18). In our study, maximum cases of children-burns were in the age group of 2–4 yr, which is consistent with the study that was reported mostly age group affected, was 0–4 yr of age group (19). Most burn patients in one study were children aged 2–6 yr old (11). Similar results were also seen in studies from some different social, such Jordan (20), Saudi Arabia (21) Turkey (22) and the UK (23). Especially development abilities greatly differ from their 2–5 yr old counterparts and likewise, the factors that place them at an increased risk. Moreover, this could be due to longer stay at home alone, high activity, and inability to protect themselves.

Our results showed that scald was the leading cause of burns (54.7%) among children-burns. Most of burning subjects between 0–10 ages were scalding, reported in a study from Arabia (21). In addition, scald was the majority cause of burns (67%) in the age group of 0–14 yr (13). The most common cause of burn injuries in children was scalded (21). While scalds were the most common type of burn injuries in children (25–28). It is due to; they may not perceive danger as properly, less control over their environment and lack of ability to escape a life-threatening burn situation. In this study, there were 38(2.4%) patients with inhalation injury, and we found electricity was the cause of 55(3.2%) of burn-related injuries. but in another study, these rates were less than ours. In similar study, there were 60 patients with inhalation injury, and electricity was the cause of 28.5% of burn-related injuries (11). In addition, 3.3% of patients were electrical injuries (17). In another study electrical burn was seen in 1.8% of patients (10). Electric burn was at 6.7% of children-burn patients (18). The number of burn injuries caused by electricity increase in children after the age of 6, owing to an increase in their activity and curiosity levels (24). Therefore, these differences in electrical-burn rate may be due to different burn patient’s size of after 6 yr old.

In the current study, most part of the body burn-affected was trunk 762 (44.4%). Moreover, burn-injuries affected the front of the body in 96% of patients (15). Patients with trunk burnt constitute the majority (45.2%) in terms of part of the body affected by burns (25). Often part of the body burn-affected was the anterior trunk (26). Children were most likely to knock over cups or pots on the table and to be splashed by the hot liquid as it falls from above.

The current study found that patients with scald-related burns had shorter hospital stay (mean hospital stay was 10.33 ± 7.12 d). In other similar study, children with scald burns usually had shorter hospital d (57.2% with hospital days less than 10 d) (11).

We found 59.9% of burn patients in our study had less than 20% TBSA. The extent of injury in the majority of children (75%) was less than 20% of the surface of the body (17). Moreover, 40.8% of children had TBSA greater than 20% (11). Extent of burn in 58% of the burn injuries was less than 21% (27). Percent of body surface burn in patients was 59% in patients with less than 20% TBSA (17). The majority of total body surface area burnt in case of children burns was less than 20% (28).

We found that the mean duration of hospitalization was 11.15 ± 8.37 d and mortality rate was 4.5%. Hospitalization and mortality in other studies have been reported 12.8 ± 9.53 and 7.2% respectively (17). Mortality rate in other study has been reported from 4.8% to 5.5% (10). 14 patients died with a percentage of 2.5% of the studied sample, was a similar study mortality rate (18). Burns mortality rate for another study was identified to be 21.3% (25). These differences may be because of different sample size. Due to the less hospital stay days and extent of the burn in our study, the hospitalization and mortality rate is desirable. In another hand, the low mortality in this study is due to dominance of mild burns that carry low mortality compared to severe burns.

There were some limitations in this study. The retrospective design meant that analysis could be done only for the data collected. It might have been appropriate and interesting to obtain data on the affected body part, and other variables such as family socioeconomic condition and educational level. Our findings may not be generalizable to the overall population of patients with children-burn injuries, therefore this is a hospital-based study conducted in a tertiary center.

## Conclusion

The majority of the patients were male with a male to female ratio of 1.7:1. Most patients were in the 2–4 yr age group with most of the injuries occurring in boys under the age of 5 yr old. It is the child’s natural curiosity and inability to understand that special things are dangerous to them, which leads to burning injury. Therefore, most affected part of the body was trunk and 1256 patients (73.2%) suffered from hot liquid burns.

## Ethical considerations

Ethical issues (Including plagiarism, informed consent, misconduct, data fabrication and/or falsification, double publication and/or submission, redundancy, etc.) have been completely observed by the authors.
